# Supplementary material for: Detection of eye contact with deep neural networks is as accurate as human experts
Source: Nat Commun. 2020 Dec 14;11:6386. doi: 10.1038/s41467-020-19712-x (PMC7736573; doi:10.1038/s41467-020-19712-x)
Supplement: Supplementary file 1 — Supplementary Information [file 41467_2020_19712_MOESM1_ESM.pdf]

Supplementary Information for "Detection of eye contact with deep neural networks is as accurate as human experts". Chong. et al.

Supplementary Information

Supplementary Table 1: Face detection accuracy analysis. True face in frame counted based on eye contact annotations (1st row), number of eye contact frames with face detection failure (2nd row), % of frames with face detection failure (3rd row). EC: eye contact.

	train	test	train+test
# of face frames with EC	281,152	25,112	306,264
# of EC frames with failed face detection	12,420	587	13,007
% of EC frames with failed face detection	4.42 %	2.34 %	4.25 %

Supplementary Table 2: Cohen's κ reliability statistics

pairs	min κ in ESCS	max κ in ESCS	average κ in ESCS	min κ in BOSCC	max κ in BOSCC	average κ in BOSCC	min κ combined	max κ combined	average κ combined
human-human	.825	.944	.886	.895	.913	.903	.825	.944	.888
human-detector	.846	.907	.880	.913	.925	.919	.846	.925	.891

Supplementary Table 3: Reliability equivalence test statistics. (*) All test cases are statistically significant at $p = .05$. m_{hc}: mean kappa scores of all human-detector pairs. m_{hh}: mean kappa scores of all human pairs.

$\Delta = 0.025$ (smallest effect size of interest).

	ESCS	BOSCC	Combined
m_{hh}	.886	.903	.888
m_{hd}	.880	.919	.891
$\text{H}_0 : m_{\text{hd}} - m_{\text{hh}} < -\Delta$	$p = 0.0347$*	$p = 0.0007$*	$p = 0.0038$*

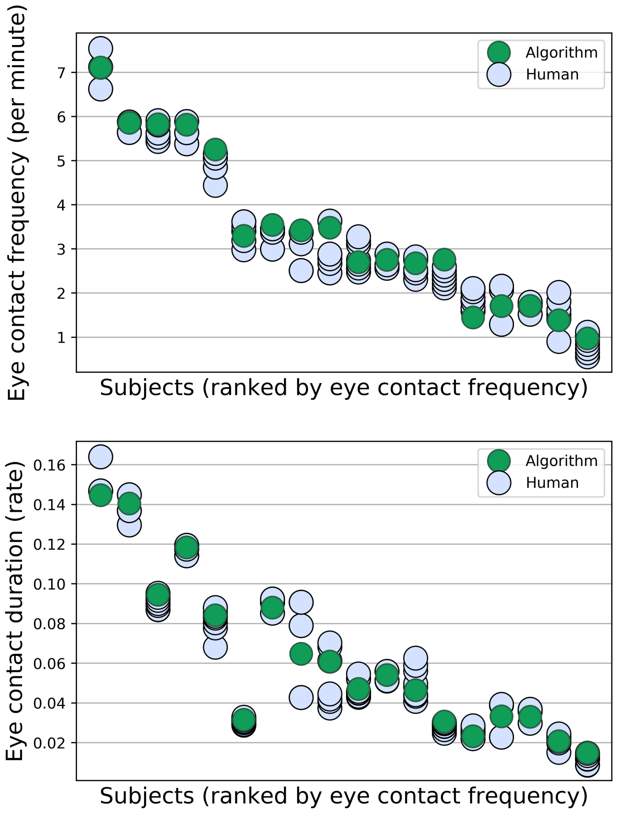

Supplementary Figure 1: Eye contact frequency (top) and duration (bottom) coded by human and algorithm for 18 validation subjects. Frequency is how many times a subject makes eye contact per minute, and duration is the length of eye contact normalized over the administration time. In both figures, the algorithm's estimate (green) is within the distribution of manual annotation (light blue). X axis is sorted by the subject's eye contact frequency such that two figures are aligned in the x axis. Subjects may make short eye contacts frequently (e.g., 3th, 6th subject) which can be also measured by the algorithm.

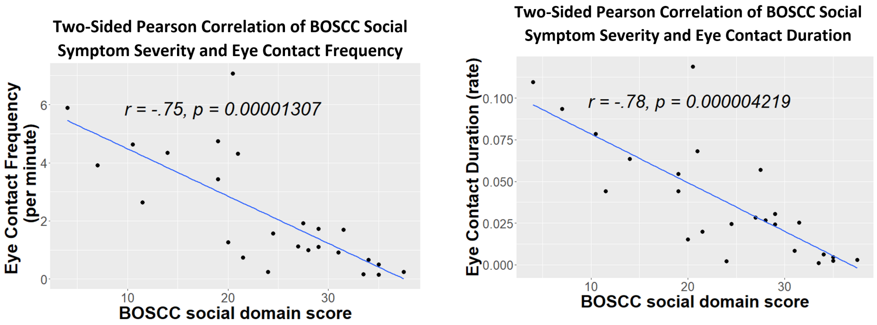

Supplementary Figure 2: Correlation between automatically measured eye contact and the severity of social impairment during the BOSCC among subjects with ASD ($n = 25$). In both frequency (left) and duration (right) are strongly negatively correlated with the severity score. Corrections were not conducted for multiple comparisons because for this set of analyses there were only 2 comparisons.

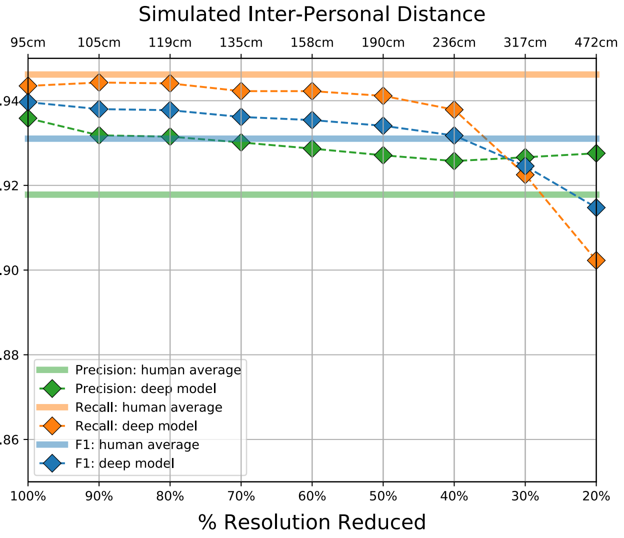

Supplementary Figure 3: We have conducted additional analysis to study the effect of varying this distance on the performance of our model. Increasing the interpersonal distance has the consequence of reducing the size of the target face in the input image. This simulation analysis is done by down-sampling the face images, to capture the presentation of faces over a range of distances. We found that the accuracy of our model is unaffected by interpersonal distances of up to 95cm (Eye contact F1 score: model = 0.940, average human = 0.932). Moreover, performance is minimally affected by distances of up to 2.3 meters (F1 score: model = 0.931). This distance range is more than adequate to cover scenarios of face-to-face interaction. For example, this range covers the zones of intimate and personal space defined in the study of proxemics (Hall. 1963) in which person-to-person social interactions occur.

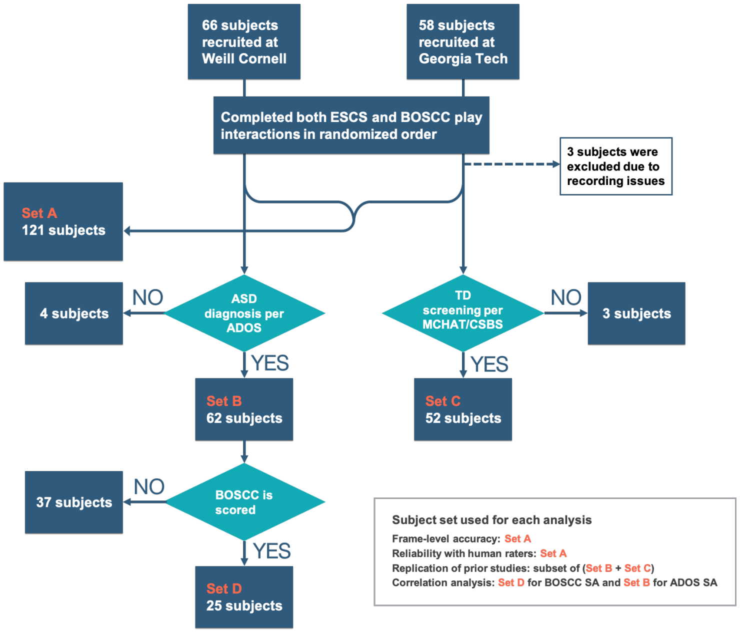

Supplementary Figure 4: Diagram illustrating inclusion/exclusion criteria of our study subjects for each analysis.
