# Supplementary material for: Detection of eye contact with deep neural networks is as accurate as human experts
Source: Nat Commun. 2020 Dec 14;11:6386. doi: 10.1038/s41467-020-19712-x (PMC7736573; doi:10.1038/s41467-020-19712-x)
Supplement: Supplementary file 3 — Reporting Summary [file 41467_2020_19712_MOESM3_ESM.pdf]

nature research

Corresponding author(s): Eunji Chong

Last updated by author(s): Oct 4, 2020

Reporting Summary

Nature Research wishes to improve the reproducibility of the work that we publish. This form provides structure for consistency and transparency in reporting. For further information on Nature Research policies, see our Editorial Policies and the Editorial Policy Checklist.

Statistics

For all statistical analyses, confirm that the following items are present in the figure legend, table legend, main text, or Methods section.

n/a	Confirmed	
[ ]	[x]	The exact sample size (n) for each experimental group/condition, given as a discrete number and unit of measurement
[ ]	[x]	A statement on whether measurements were taken from distinct samples or whether the same sample was measured repeatedly
[ ]	[x]	The statistical test(s) used AND whether they are one- or two-sided<br>*Only common tests should be described solely by name; describe more complex techniques in the Methods section.*
[ ]	[x]	A description of all covariates tested
[x]	[ ]	A description of any assumptions or corrections, such as tests of normality and adjustment for multiple comparisons
[ ]	[x]	A full description of the statistical parameters including central tendency (e.g. means) or other basic estimates (e.g. regression coefficient) AND variation (e.g. standard deviation) or associated estimates of uncertainty (e.g. confidence intervals)
[ ]	[x]	For null hypothesis testing, the test statistic (e.g. F, t, r) with confidence intervals, effect sizes, degrees of freedom and P value noted<br>*Give P values as exact values whenever suitable.*
[x]	[ ]	For Bayesian analysis, information on the choice of priors and Markov chain Monte Carlo settings
[x]	[ ]	For hierarchical and complex designs, identification of the appropriate level for tests and full reporting of outcomes
[ ]	[x]	Estimates of effect sizes (e.g. Cohen's d, Pearson's r), indicating how they were calculated

Our web collection on statistics for biologists contains articles on many of the points above.

Software and code

Policy information about availability of computer code

Data collection	Commercial software - Mangold International's INTERACT (Version 17) - was used to organize and annotate the data.
Data analysis	Custom code written in Python (Version 2.7) was used, which is available at https://github.com/rehg-lab/eye-contact-cnn.

For manuscripts utilizing custom algorithms or software that are central to the research but not yet described in published literature, software must be made available to editors and reviewers. We strongly encourage code deposition in a community repository (e.g. GitHub). See the Nature Research guidelines for submitting code & software for further information.

Data

Policy information about availability of data

All manuscripts must include a data availability statement. This statement should provide the following information, where applicable:

- Accession codes, unique identifiers, or web links for publicly available datasets
- A list of figures that have associated raw data
- A description of any restrictions on data availability

Three datasets that were used in the first stage of training are publicly available;
MPIIFaceGaze: https://www.mpi-inf.mpg.de/departments/computervision-and-machine-learning/research/gaze-based-human-computer-interaction/its-written-all-over-your-face-full-face-appearance-based-gaze-estimation.
EYEDIAP: https://www.idiap.ch/dataset/eyediap.
SynHead:https://research.nvidia.com/publication/dynamic-facial-analysis-bayesianfiltering-recurrent-neural-networks.
The IRB protocol for this project prohibits the release of the eye contact dataset itself.

Field-specific reporting

Please select the one below that is the best fit for your research. If you are not sure, read the appropriate sections before making your selection.

☐ Life sciences ☒ Behavioural & social sciences ☐ Ecological, evolutionary & environmental sciences

For a reference copy of the document with all sections, see nature.com/documents/nr-reporting-summary-flat.pdf

Behavioural & social sciences study design

All studies must disclose on these points even when the disclosure is negative.

Study description	Quantitative cross-sectional.<br>From a sample of 66 children with Autism Spectrum Disorder and 58 typically developing children (18-60 months), video recordings of two semi-structured play interactions were collected and used to train an automatic eye contact classifier.
Research sample	Sample of 121 young children (66 with autism diagnosis, 91 male, age M = 36.5 months) were used in the main analysis. For replication analysis, sample from Jones, R. M. et al. 2017. was used.<br>The sample was chosen to train and test models using both a group of children representative of the general population (typically-developing group), as well as children for whom eye contact is a clinically-meaningful behavior that researchers and clinicians are interested in measuring (ASD group). The included age group (18-60 months) was selected as non-verbal means of communication such as eye contact is especially important at this age as children are still developing language. The sample includes more boys than girls because current estimates suggest that the ratio of boys with ASD diagnoses to girls with ASD diagnoses is 3:1 (Loomes et al., 2017), and the typically-developing group also includes more boys than girls so that the gender distribution was relatively even across groups in the study.
Sampling strategy	Convenience sampling was used, in that all participants who were interested in participating in the study and who met study inclusion criteria were included.<br>Previous work on the topic of automated eye contact detection (Ye, Z. et al. 2015, Chong, E. et al. 2017) suggest that it is possible to successfully train a model with a sample size much smaller than ours. Therefore our chosen sample size can be considered sufficient.
Data collection	A stationary camcorder mounted on a tripod and a wearable point-of-view camera were used to record the data.<br>Participant's parent was present at times during data collection.<br>There were no experimental conditions; researchers were not blind to study hypothesis however there was no experimental manipulation.
Timing	The data was collected between 2015 and 2018.
Data exclusions	No data were excluded from the analysis altogether. Data from n=7 subjects were excluded from analyses comparing groups because of unclear diagnostic categorization.
Non-participation	No participants dropped out/declined participation.
Randomization	Participants were not allocated into experimental groups.<br>Covariates were not controlled, as the focus of the study was not on group comparison.

Reporting for specific materials, systems and methods

We require information from authors about some types of materials, experimental systems and methods used in many studies. Here, indicate whether each material, system or method listed is relevant to your study. If you are not sure if a list item applies to your research, read the appropriate section before selecting a response.

Materials & experimental systems

n/a	Involved in the study	
☒	☐	Antibodies
☒	☐	Eukaryotic cell lines
☒	☐	Palaeontology and archaeology
☒	☐	Animals and other organisms
☐	☒	Human research participants
☒	☐	Clinical data
☒	☐	Dual use research of concern

Methods

n/a	Involved in the study	
☒	☐	ChIP-seq
☒	☐	Flow cytometry
☒	☐	MRI-based neuroimaging

Human research participants

Policy information about studies involving human research participants

Population characteristics	See above.
Recruitment	Participants with autism were recruited by contacting families who participated in research or clinical services at our center previously and had indicated that they would like to be contacted about future research projects. Typically-developing participants were recruited from the community, by posting flyers in community locations such as pediatrician offices. Interested participants then contacted the research team. Across both groups, therefore, participants are those who are interested in participating in research, however there is no literature to indicate that the extent to which a family is interested in participating in research would be related to their child's use of eye contact in any way, and therefore it is unlikely that this recruitment method impacted results.
Ethics oversight	Georgia Institute of Technology. Weill Cornell Medicine.

Note that full information on the approval of the study protocol must also be provided in the manuscript.
